# Brief counselling after home‐based HIV counselling and testing strongly increases linkage to care: a cluster‐randomized trial in Uganda

**DOI:** 10.1002/jia2.25014

**Published:** 2017-10-20

**Authors:** Eugene Ruzagira, Heiner Grosskurth, Anatoli Kamali, Kathy Baisley

**Affiliations:** ^1^ Department of Infectious Disease Epidemiology London School of Hygiene and Tropical Medicine London UK; ^2^ MRC/UVRI Uganda Research Unit on AIDS Entebbe Uganda; ^3^ International AIDS Vaccine Initiative New York USA

**Keywords:** HIV/AIDS, home‐based HIV counselling and testing, linkage to care, Uganda, Africa

## Abstract

**Introduction:**

The aim of this study was to determine whether counselling provided subsequent to HIV testing and referral for care increases linkage to care among HIV‐positive persons identified through home‐based HIV counselling and testing (HBHCT) in Masaka, Uganda.

**Methods:**

The study was an open‐label cluster‐randomized trial. 28 rural communities were randomly allocated (1:1) to intervention (HBHCT, referral and counselling at one and two months) or control (HBHCT and referral only). HIV‐positive care‐naïve adults (≥18 years) were enrolled. To conceal participants’ HIV status, one HIV‐negative person was recruited for every three HIV‐positive participants. Primary outcomes were linkage to care (clinic‐verified registration for care) status at six months, and time to linkage. Primary analyses were intention‐to‐treat using random effects logistic regression or Cox regression with shared frailty, as appropriate.

**Results:**

Three hundred and two(intervention, *n* = 149; control, *n* = 153) HIV‐positive participants were enrolled. Except for travel time to the nearest HIV clinic, baseline participant characteristics were generally balanced between trial arms. Retention was similar across trial arms (92% overall). One hundred and twenty‐seven (42.1%) participants linked to care: 76 (51.0%) in the intervention arm *versus* 51 (33.3%) in the control arm [odds ratio = 2.18, 95% confidence interval (CI) = 1.26–3.78; *p* = 0.008)]. There was evidence of interaction between trial arm and follow‐up time (*p* = 0.009). The probability of linkage to care, did not differ between arms in the first two months of follow‐up, but was subsequently higher in the intervention arm *versus* the control arm [hazard ratio = 4.87, 95% CI = 1.79–13.27, *p* = 0.002].

**Conclusions:**

Counselling substantially increases linkage to care among HIV‐positive adults identified through HBHCT and may enhance efforts to increase antiretroviral therapy coverage in sub‐Saharan Africa.

## 1 Introduction

Home‐based HIV counselling and testing (HBHCT) is highly acceptable 1, 2, 3 and has the potential to substantially increase knowledge of HIV status in sub‐Saharan Africa (SSA) 4. However, linkage to care following HBHCT is often inadequate. In the absence of interventions to facilitate linkage, less than one‐third of HIV‐positive persons identified through HBHCT in SSA link to care 5.

In order for the populations targeted by HBHCT to benefit from HIV prevention and care services, interventions that can link them to these services need to be identified and utilized. Results from observational studies suggest that counselling provided after referral could increase linkage to care among HIV‐positive persons identified through HBHCT in SSA 6, 7, 8, 9, 10. A major limitation of observational studies, however, is that it is difficult to account for the effects of confounding factors. Therefore, randomized trials are required to determine the potential effects of counselling on linkage to care.

In this study, we evaluated the effectiveness of counselling after HIV diagnosis and referral to care, compared to referral to care only, on linkage to care among HIV‐positive individuals identified through HBHCT in Masaka, Uganda. We used a cluster‐randomized design to reduce the risk of contamination between trial arms and increase acceptability of the interventions.

## 2 Methods

### 2.1 Study design and participants

The study was an open‐label cluster‐randomized controlled trial. A description of the methods has been previously reported 11. Briefly, 28 communities (clusters) were randomly allocated (1:1) to intervention (HBHCT, referral and counselling at one and two months) or control (HBHCT and referral only). HBHCT was offered to all adults (≥18 years). Newly and previously identified HIV‐positive persons were eligible to participate if they were able to consent, not previously or currently in care, available for follow‐up, and not participating in other health‐related research. To reduce the possibility of revealing participants’ HIV status to other community members, one HIV‐negative adult was recruited for every three HIV‐positive participants.

### 2.2 Study setting

The study area comprised three rural subcounties of Masaka, a district in south‐western Uganda. The subcounties have clinics that offer free HIV care and antiretroviral therapy (ART). Typically, patients newly presenting for care were issued a clinic number and card (registration), underwent a clinical examination and CD4 count testing (results were usually available after two to four weeks), started on cotrimoxazole prophylaxis (CTXp), and if eligible, ART. At the time of the study, the CD4 count threshold for ART eligibility in Uganda was ≤500 cells/mm^3^ 12.

### 2.3 Intervention

#### 2.3.1 Development

The intervention was developed based on published evidence indicating that psychosocial factors are common barriers of linkage to HIV care in SSA 13, 14, 15, 16, 17, 18, 19, 20, 21, 22, 23, and that counselling can reduce the effects of these barriers 17, 24, 25, 26, 27, 28 and may increase linkage 6, 7, 9.

#### 2.3.2 Content

Counselling sessions addressed the following generic points: acceptance of HIV diagnosis, plans to seek care, fear or experience of stigma, importance of HIV status disclosure and availability of psychosocial support for linkage to care; and information about available care services, antiretroviral drugs and the rationale for early linkage to care. In addition, the sessions were used to address personal issues, e.g. marital discord arising after disclosure of HIV status. HIV‐negative participants in the intervention arm received HIV risk reduction counselling and information on the importance of regular HCT.

#### 2.3.3 Staff training

Counsellors did not have medical training but were trained in HCT and had experience in conducting community‐based HCT. Training on the intervention was conducted in the four weeks prior to study initiation and consisted of: i) one group seminar that comprised a didactic 30‐min presentation and 60‐min interactive session; ii) a single 30‐min one‐on‐one session with each of the counsellors that was conducted one to two weeks after the seminar.

#### 2.3.4 Quality assurance

Counsellors were provided with a checklist of the counselling content to refer to during the sessions. In addition, counsellors took notes of the issues discussed in each session. The study investigator reviewed these notes and discussed any omissions with individual counsellors.

### 2.4 Outcomes

Primary outcomes were linkage to care (clinic‐verified registration for care), determined six months after HIV diagnosis, and time to linkage. Secondary outcomes were time to clinic‐verified receipt of a CD4 count and ART initiation, and self‐reported adherence to CTXp. A further secondary outcome was uptake of repeat HCT at the six month visit among HIV‐negative participants.

### 2.5 Randomization

A cluster was defined as a village or a set of villages with at least 400 adults. Clusters were separated by a buffer zone of ≥1 non‐participating villages to minimize the risk of contamination. Clusters were randomly allocated to intervention and control arms. Stratification and restricted randomization were used to minimize between‐cluster variation and achieve overall balance between trial arms with regard to key variables 11. Stratification was defined by distance (≤10 or >10 km) from the district capital, and by cluster make‐up, i.e. whether the cluster was composed of a single village or several villages. Restricted randomization was then applied to achieve balance on the following cluster‐level variables: size (total number of adults); presence of a trading centre; location along a major road; lakeshore location and presence of an HIV clinic within 5 km.

### 2.6 Procedures

#### 2.6.1 HCT

Following community mobilization, standard HCT (including pre‐test and post‐test counselling) was provided to all adults in each randomized community. Married/cohabiting individuals had the option of receiving HCT as a couple. Blood obtained by finger‐prick was tested using HIV rapid test kits: Alere Determine HIV‐1/HIV‐2 (Alere Medical, Japan) for screening, Stat‐Pak HIV 1/2 (Chembio Diagnostic systems, USA) for confirmation of positive results and Uni‐Gold HIV 1/2 (Trinity Biotech, Ireland) as tie‐breaker. Post‐test counselling included a discussion of HIV risk reduction strategies, disclosure plans, partner testing, care and support services and referral options.

#### 2.6.2 Enrolment

Eligible individuals received information about the study including that their community could be in either of the trial arms and invited to consent. After enrolment, individuals were informed about which trial arm their community was allocated to. Participants completed a counsellor‐administered questionnaire on socio‐demographic characteristics and HCT history.

#### 2.6.3 Referral

HIV‐positive participants received a referral form a copy of which was retained in a centrally stored folder. Data recorded on the form included the participant's names, age, sex, study number and address.

#### 2.6.4 Counselling sessions

These were conducted at participants’ homes one and two months after HBHCT; each lasted approximately 45 min. Counselling was conducted using a client‐centred approach, i.e. it was tailored to the participant's needs and circumstances 29. Issues identified in the first session were followed up in the second session (for 92% of the participants, both sessions were conducted by the same counsellor). Counsellors worked across clusters.

#### 2.6.5 Collection of outcome data

Six months after HBHCT, all participants were visited by a counsellor (for 58% of the participants in the intervention arm, this was the same counsellor who had conducted the counselling sessions) to collect information on study outcomes. To assess the potential of contamination between trial arms, counsellors asked participants if they had discussed the study with fellow participants from other villages.

For each participant who reported that they linked to care, a field team member visited the clinic to verify whether the participant had indeed been registered for care. Participants whose records could not be found were re‐interviewed to clarify whether they had actually linked or not. Only participants whose records were found were categorized as “linkers”. Participants’ clinic records were checked to confirm CD4 count testing and receipt of the results, and initiation of CTXp and ART. Adherence to CTXp was measured by self‐reported responses to a question on missed doses. Participants who missed ≤5 of 30 doses in the past month were categorized as “adherers”.

#### 2.6.6 HIV‐negative study component

Similar enrolment and follow‐up counselling procedures were applied to HIV‐negative participants. Repeat HBHCT was offered to participants in both arms at six months.

### 2.7 Ethical considerations

The trial was approved by Uganda Virus Research Institute Research Ethics Committee, the Uganda National Council for Science and Technology, and the Ethics Committee of the London School of Hygiene and Tropical Medicine. Written informed consent was obtained from each participant. The trial is registered at ClinicalTrials.gov (NCT02497456).

### 2.8 Sample size

On the basis of findings from HBHCT studies in which only referral was provided following HIV diagnosis 30, 31, 32, 33 and those in which counselling was provided after referral 6, 7, we assumed linkage of 35% in the control arm and that this would increase to 60% in the intervention arm. We aimed to have 80% power to detect this increase at a significance level of 5%. On the basis of data from settings similar to Masaka 11, we assumed that the between‐cluster coefficient of variation (*k*) for linkage in the absence of intervention was 0.25. Based on these assumptions and an estimated harmonic mean of seven participants per cluster, 28 clusters would be needed. This sample size would also provide >80% power to detect a hazard ratio of 1.7 for the effect of the intervention on time to linkage 34.

### 2.9 Analysis

All analyses were pre‐specified. The primary analyses were intention‐to‐treat and based on individual‐level data, since there was a sufficient number of clusters per arm and the cluster size varied considerably 35. Random effects logistic regression and Cox regression with shared frailty were used to estimate the effect of the intervention on linkage and time to linkage respectively. Participants who were lost to follow‐up were assumed not to have linked. Those who were reported at the six month follow‐up visit to have moved were censored midway between enrolment and that visit. Those who were in the study area but did not attend the six month visit were censored at that visit. The primary analyses of the intervention effect were adjusted for randomization stratum as a fixed effect. Secondary analyses were adjusted for age and sex *a priori*, and other characteristics that showed substantial baseline imbalance. Due to the nature of the intervention (repeated counselling), it was expected that its effect might change over time. Therefore, the proportional hazards assumption was examined by splitting follow‐up time into two intervals (0–2 months and >two months) at a point corresponding with the time of the second counselling session, and testing for an interaction between trial arm and time. Similar methods were used to estimate the effect of the intervention on the proportions that adhered to CTXp, and on the time to obtaining CD4 counts, and ART initiation among HIV‐positive participants, and on the proportion that accepted repeat HCT among HIV‐negative participants.

Analyses based on cluster‐level summaries (Supplementary File) were also performed to check the robustness of the individual‐level analysis, using methods for stratified cluster‐randomized trials 36. Intervention effects on binary outcomes were measured using prevalence ratios (PR), calculated as the ratio of the arithmetic mean of the cluster‐specific prevalence of the outcome in each arm. The 95% confidence interval (CI) was calculated with variance estimated from the residual mean square from a two‐way analysis of variance (ANOVA) of cluster‐specific prevalence on stratum and trial arm. Adjusted PR were calculated as the arithmetic mean ratio of observed to expected prevalences in each cluster, with logistic regression used to estimate the expected prevalence, adjusted for age, sex, stratum and other variables that showed baseline imbalance. CIs were obtained from an ANOVA of the observed/expected prevalences on stratum and trial arm, as described above. Intervention effects on time‐to‐event outcomes were measured using rate ratios (RR) by similar methods; Poisson regression was used to estimate expected number of events for the adjusted analyses.

Data from the control arm were used to estimate *k* for linkage to care. STATA version 12.0 (StataCorp, College Station, TX, USA) was used for all analyses.

## 3 Results

**Figure 1:**
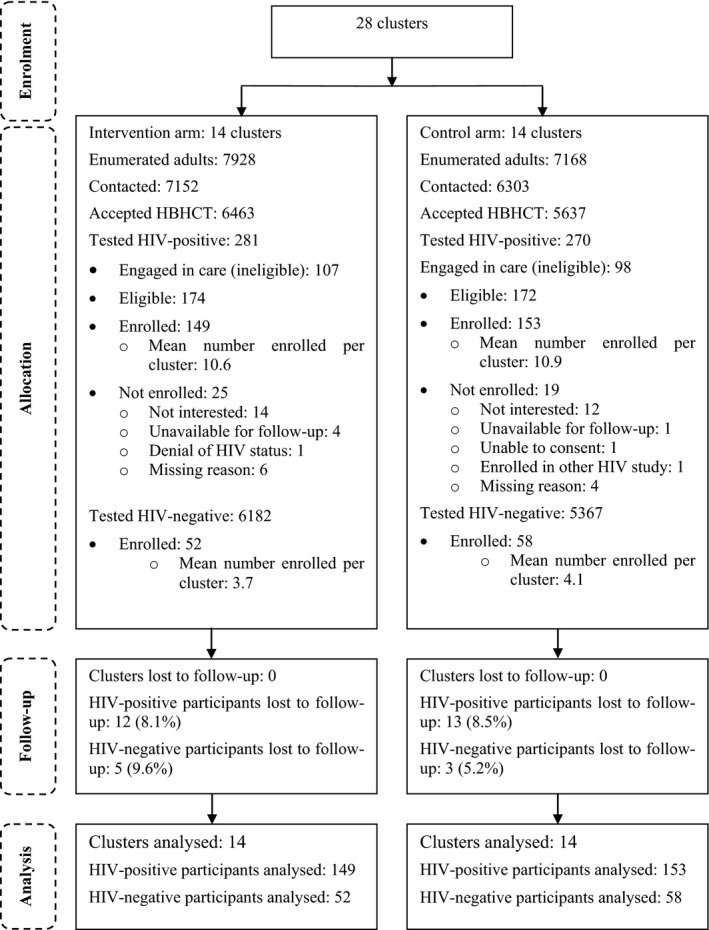
Flow of clusters and participants through the trial.

The study was conducted between March 2015 and March 2016. 13,455 people (89.1% of those enumerated) were contacted of which 12,100 (89.9%) accepted HBHCT (Figure 1). Common reasons for declining HBHCT were being HIV‐positive and already engaged in care (437, 32.3%), not wanting to know one's HIV status (397, 29.3%), and having recently undergone HCT (214, 15.8%). Of those who accepted HBHCT, 551 (4.6%) tested HIV‐positive, of whom 205 (37.2%) were already in care and thus ineligible. Of those eligible, 302 (87.3%) were enrolled, of whom 265 (87.7%) were newly diagnosed with HIV. 110 HIV‐negative individuals were also enrolled.

The median age of HIV‐positive participants was 30.0 years (interquartile range, 25.0–39.0; Table 1); the majority were female (54.6%), married/cohabiting (60.3%), had incomplete primary school/no formal education (60.3%) and had previously undergone HCT (80.5%). Baseline variables were generally balanced between trial arms; however, participants in the intervention arm were more likely to report <30 min travel time to the HIV clinic than those in the control arm (34.9% vs. 22.2%). 25 (8.3%) HIV‐positive participants were lost to follow‐up: 13 (4.3%) moved; 8 (2.6%) for unknown reasons; 2 (<1%) died; and 2 (<1%) withdrew. The distribution of baseline characteristics among HIV‐negative participants was similar to that of HIV‐positive participants (Table 1). 8 (7.3%) were lost to follow‐up.

**Table 1:** Baseline characteristics of participants

	HIV‐positive participants		HIV‐negative participants	
	Intervention	Control	Intervention	Control
	N (%)	N (%)	N (%)	N (%)
Total enrolled	149	153	52	58
Sex				
Female	76 (51.0)	89 (58.2)	26 (50.0)	37 (63.8)
Male	73 (49.0)	64 (41.8)	26 (50.0)	21 (36.2)
Median age in years (IQR)	30.0 (25.0–38.0)	30.0 (25.0–40.0)	35.0 (25.0–44.5)	31.5 (23.0–42.0)
Age group				
18–24 years	34 (22.8)	37 (24.2)	10 (19.2)	16 (27.6)
25–34 years	63 (42.3)	58 (37.9)	15 (28.9)	18 (31.0)
35–44 years	29 (19.5)	30 (19.6)	14 (26.9)	13 (22.4)
45+ years	23 (15.4)	28 (18.3)	13 (25.0)	11 (19.0)
Marital status				
Married/cohabiting	85 (57.1)	97 (63.4)	38 (73.1)	43 (74.1)
Single	22 (14.8)	26 (17.0)	6 (11.5)	10 (17.2)
Divorced/separated/widowed	42 (28.2)	30 (19.6)	8 (15.4)	5 (8.6)
Education				
None/incomplete primary	86 (57.7)	96 (62.8)	22 (42.3)	25 (43.1)
Primary	34 (22.8)	32 (20.9)	9 (17.3)	14 (24.1)
Above primary	29 (19.5)	25 (16.3)	21 (40.4)	19 (32.8)
Occupation				
Subsistence farmer	84 (56.4)	80 (52.3)	16 (30.8)	25 (43.1)
Other	65 (43.6)	73 (47.7)	36 (69.2)	33 (56.9)
Socio‐economic status^a^				
Low	56 (37.6)	61 (39.9)	10 (19.2)	19 (32.8)
Middle	52 (34.9)	56 (36.6)	18 (34.6)	14 (24.1)
High	41 (27.5)	36 (23.5)	24 (46.2)	25 (43.1)
Travel time to nearest HIV clinic				
<30 minutes	52 (34.9)	34 (22.2)	21 (40.4)	12 (20.7)
30 minutes or more	97 (65.1)	119 (77.8)	31 (59.6)	46 (79.3)
Ever tested for HIV				
No	30 (20.1)	29 (19.0)	1 (1.9)	1 (1.7)
Yes	119 (79.9)	124 (81.0)	51 (98.1)	57 (98.3)
Tested for HIV in the last 12 months				
No	71 (47.7)	81 (52.9)	6 (11.5)	5 (8.6)
Yes	78 (52.4)	72 (47.1)	46 (88.5)	53 (91.4)
Previously aware of HIV‐positive status				
No	131 (87.9)	134 (87.6)	–	–
Yes	18 (12.1)	19 (12.4)	–	–

IQR, interquartile range.

a Socio‐economic status categories were obtained from a wealth index scale based on ownership of household and other properties using principal component analysis.

### 3.1 Linkage to care

134/302 participants (44.4%) reported to have linked to care but clinic records were found for 127 (42.1%) only. Seven participants whose clinic records could not be found were re‐interviewed; all admitted that they had not linked. Of those confirmed to have linked, 8 (6.3%) had registered with a facility outside of the study area. Linkage to care was higher in the intervention arm compared to the control arm [51.0% *versus* 33.3%; odds ratio (OR) = 2.18, 95% CI = 1.26–3.78; Table 2]. The effect of the intervention was similar after adjusting for age, sex and travel time to the clinic [adjusted (aOR = 2.14, 95% CI = 1.24–3.7)]. The crude and adjusted effect estimates from the cluster‐level analysis were consistent with a higher linkage in the intervention *versus* the control arm [prevalence ratio (PR)  = 1.59, 95% C = 1.09–2.33; aPR = 1.58, 95% CI = 1.07–2.34].

**Table 2:** Effect of follow‐up counselling on linkage to care, adherence to cotrimoxazole prophylaxis (CTXp) and uptake of repeat HCT

	Intervention arm	Control arm	OR (95% CI)	*p*‐value	aOR (95% CI)^a^	*p*‐value
Among HIV‐positive participants						
Linkage to care	76/149 (51.0)	51/153 (33.3)	2.18 (1.26–3.78)	0.008	2.14 (1.24–3.70)	0.009
Adherence to CTXp	66/149 (44.3)	43/153 (28.1)	2.15 (1.16–3.98)	0.02	2.17 (1.20–3.93)	0.01
Among HIV‐negative participants						
Uptake of repeat HIV test	42/52 (80.8)	46/58 (79.3)	1.08 (0.42–2.78)	0.87	0.70 (0.24–2.03)	0.52

OR, odds ratio; aOR, adjusted odds ratio; CI, confidence interval.

a Adjusted for age, sex, strata and travel time to the nearest HIV clinic.

**Figure 2:**
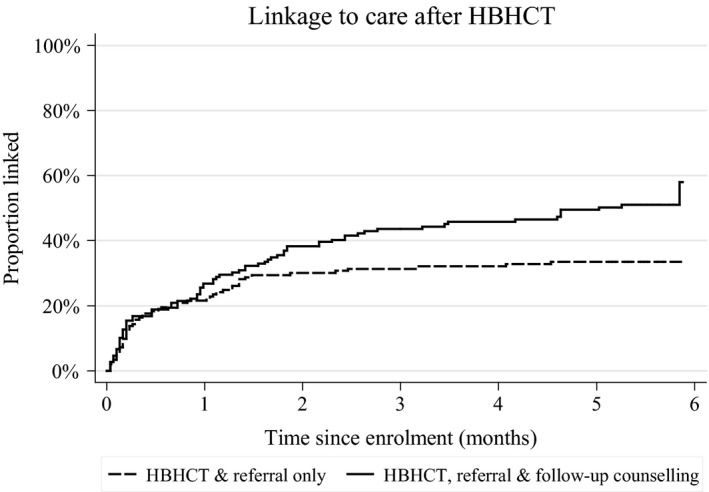
Kaplan–Meier estimates of linkage to care.

The probability of linkage was similar in both trial arms up to the first month of follow‐up. Subsequently, more participants linked in the intervention arm than in the control arm with the difference becoming more marked around the second month of follow‐up (Figure 2). The overall hazard ratio (HR) was 1.65 (95% CI = 1.11–2.44) and was similar after adjusting for age, sex and travel time to the HIV clinic [adjusted hazard ratio (aHR) = 1.62, 95% CI = 1.12 to 2.33; Table 3]. There was strong evidence of interaction between trial arm and follow‐up time (*p* = 0.009). In the first interval (0–2 months), 57 (38.3%) participants linked in the intervention arm *versus* 46 (30.1%) in the control arm [HR = 1.32, 95% CI = 0.86–2.03; aHR = 1.30, 95% CI = 0.87–1.94]. In the second interval (>two months), 19 (20.7%) participants linked in the intervention arm *versus* 5 (4.7%) in the control arm [HR = 4.87, 95% CI = 1.79–13.27; aHR = 4.78, 95% CI = 1.77–12.89]. The cluster‐level analysis was also consistent with an intervention effect on rate of linkage [rate ratio (RR) = 1.84, 95% CI = 0.99–3.42; aRR = 1.92, 95% CI = 0.96–3.86)].

**Table 3:** Effect of follow‐up counselling on time to linkage to care, obtaining CD4 counts and antiretroviral therapy (ART) initiation

	Intervention arm			Control arm			HR (95% CI)	*p*‐value	aHR (95% CI)^a^	*p*‐value
	N	*n*	pm	N	*n*	pm				
Linkage to care^b^										
Entire follow‐up period	149	76	492	153	51	590	1.65 (1.11–2.44)	0.02	1.62 (1.12–2.33)	0.02
0–2 months	149	57	224	153	46	239	1.32 (0.86–2.03)	0.20	1.30 (0.87–1.94)	0.20
>2 months	92	19	268	107	5	351	4.87 (1.79–13.27)	0.002	4.78 (1.77–12.89)	0.002
Obtaining CD4 counts^b^										
Entire follow‐up period	149	67	561	153	40	664	1.91 (1.25–2.93)	0.005	1.86 (1.23–2.80)	0.007
0–2 months	149	40	247	153	30	266	1.45 (0.88–2.40)	0.14	1.41 (0.87–2.28)	0.17
>2 months	109	27	313	123	10	398	3.35 (1.59–7.04)	0.001	3.27 (1.57–6.81)	0.002
ART initiation^b^										
Entire follow‐up period	149	50	630	153	40	662	1.31 (0.85–2.04)	0.22	1.33 (0.85–2.06)	0.21
0–2 months	149	25	264	153	33	262	0.78 (0.46–1.34)	0.37	0.79 (0.46–1.34)	0.38
>2 months	124	25	365	120	7	399	3.90 (1.67–9.11)	0.002	3.96 (1.69–9.26)	0.002

N, sample size; *n*, number with outcome; pm, person‐months; HR, hazard ratio; aHR, adjusted hazard ratio; CI, confidence interval.

a Adjusted for age, sex, strata and travel time to nearest HIV clinic.

b The respective *p*‐values for interaction between trial arm and follow‐up time for linkage to care, obtaining CD4 counts and ART initiation were 0.009, 0.05 and 0.0007.

### 3.2 Obtaining CD4 counts

One hundred and seven (35.4%) participants obtained CD4 counts; 67 (45.0%) in the intervention arm *versus* 40 (26.1%) in the control arm. The overall HR was 1.91 (95% CI = 1.25–2.93; Table 3) and was similar after adjusting for age, sex and travel time to the HIV clinic [aHR = 1.86, 95% CI = 1.23–2.80]. There was some evidence of interaction between trial arm and follow‐up time (*p* = 0.05). In the first follow‐up interval, 40 (26.8%) participants obtained CD4 counts in the intervention arm *versus* 30 (19.6%) in the control arm [HR = 1.45, 95% CI = 0.88–2.40; aHR = 1.41, 95% CI = 0.87–2.28]. In the second follow‐up interval, 27 (24.8%) participants obtained CD4 counts in the intervention arm *versus* 10 (8.1%) in the control arm [HR = 3.35, 95% CI = 1.59–7.04; aHR=3.27, 95% CI = 1.57–6.81]. Results from the cluster‐level analysis were consistent with an intervention effect on time to obtaining CD4 counts [RR = 2.10, 95% CI = 1.14–3.84; aRR = 2.12, 95% CI = 1.22–3.70].

### 3.3 ART initiation

Of those enrolled, 90 (29.8% of all participants and 70.9% of those who linked) individuals initiated ART. This included 61 (95.3%) of 64 individuals who had a CD4 count of ≤500 cells/mm^3^, the ART eligibility threshold at the time of the study 12. Overall, 50 (33.6%) participants initiated ART in the intervention arm *versus* 40 (26.1%) in the control arm [HR = 1.31 (95% CI = 0.85–2.04)]. The effect estimate was unchanged after adjusting for age, sex and travel time to the HIV clinic [aHR = 1.33, 95% CI = 0.85–2.06; Table 3]. There was strong evidence of interaction between trial arm and follow‐up time (*p* = 0.0007). In the first follow‐up interval, 25 (16.8%) participants initiated ART in the intervention arm *versus* 33 (21.6%) in the control arm (HR = 0.78, 95% CI = 0.46–1.34; aHR = 0.79, 95% CI = 0.46–1.34). In the second follow‐up interval, 25 (20.2%) participants initiated ART in the intervention arm *versus* 7 (5.8%) in the control arm (HR = 3.90, 95% CI = 1.67–9.11; aHR = 3.96, 95% CI = 1.69–9.26). In the cluster‐level analysis, the intervention also increased ART initiation overall, but there was no evidence of a significant difference (RR = 1.21, 95% CI = 0.69–2.13; aRR = 1.27, 95% CI = 0.77–2.12).

### 3.4 Adherence to CTXp

36.1% of the participants reported adhering to CTXp; adherence was higher in the intervention arm compared to the control arm [44.3% *versus* 28.1%; OR = 2.15, 95% CI = 1.16–3.98; adjusted odds ratio (aOR) (adjusted for age, sex and time to the HIV clinic) = 2.17, 95% CI = 1.20–3.93; Table 2]. The cluster‐level estimates for the effect of intervention on adherence to CTXp were also consistent with significantly higher adherence in the intervention arm compared to the control arm (PR = 1.69 95% CI = 1.04–2.75; aPR = 1.70, 95% CI = 1.06–2.74).

### 3.5 Uptake of repeat HCT among HIV‐negative participants

Overall uptake of repeat HCT was 80.0%. There was no evidence of a difference in uptake between intervention and control arms [80.8% *versus* 79.3%; OR = 1.08, 95% CI = 0.42–2.78; aOR (adjusted for age, sex and time to the HIV clinic) = 0.70, 95% CI = 0.24–2.03; Table 2]. Conclusions from the cluster‐level analysis were similar (PR = 0.95, 95% CI = 0.76–1.20; aPR = 0.90, 95% CI = 0.72–1.13).

### 3.6 Contamination

Two (<1%) individuals reported that they had discussed the trial with participants from villages other than their own.

### 3.7 Coefficient of variation

*k* values for linkage to care and rate of linkage were 0.33 and 0.52 respectively.

## 4 Discussion

This trial showed that an intervention comprising two brief counselling sessions for adults identified with HIV through HBHCT strongly increases linkage to care. In the individual‐level analysis, the intervention was associated with a twofold increase in the proportion of persons linking to care, and approximately 5‐fold increase in the hazard of linkage after the second counselling visit; the estimates did not change after adjusting for age, sex and travel time to the clinic. Results from the cluster‐level analysis were also consistent with an intervention effect on linkage to care and time to linkage. The trial also showed that counselling significantly increased the rate of obtaining CD4 counts and ART initiation, and adherence to CTXp. For all time‐to‐event outcomes, the intervention effect increased with time, and became apparent after the second counselling visit. Although the two counselling sessions covered similar content, the second session also provided an opportunity to follow‐up on issues identified in the first session as well as address new concerns.

The effect estimates observed in our trial were stronger than those observed in the only other trial that previously investigated the impact of counselling on linkage to care among HIV‐positive persons identified through community‐based HCT (mobile HCT and HBHCT) in SSA 37. Counselling in that trial increased the proportion of individuals linking to care by 4% only. This was probably due to the already high (89%) level of linkage to care in the standard‐of‐care (referral only) arm of that trial. In contrast, linkage to care in our standard‐of‐care arm was 33%, a figure consistent with that observed after routine referral in previous studies 20, 30, 31, 38, 39, 40.

Linkage to care did not differ between trial arms in the month between HIV diagnosis and the first counselling session, suggesting that on its own, knowledge of trial arm had no effect on linkage. The probability of linking to care was also higher in the first month than in subsequent periods. This has been observed previously 10, 38 and may mean that persons who accept their status, and are motivated to get help, promptly link to care following HIV diagnosis 41. The finding also suggests that one month after HIV diagnosis may be the optimal time for targeting persons who are less motivated to seek care.

Whilst the overall proportion of participants who initiated ART was low, ART initiation among those who linked was high. This suggests that linkage to care is the main bottleneck to uptake of ART in this population. As the new WHO guidelines recommending ART initiation regardless of CD4 count become widely adopted, the focus of linkage efforts is likely to shift from just registering for care to ART initiation 42. Even so, in the absence of home‐based ART initiation, HIV‐positive patients identified through HBHCT will still have to visit and register with an HIV clinic before receiving ART.

Although linkage to care was significantly higher in the intervention arm, it was still low 25. This finding points to the existence of additional barriers that cannot be addressed by counselling and the need to identify and evaluate interventions that target these barriers. Such barriers may be programmatic; socio‐cultural or structural 24. Since no single intervention is likely to address all linkage barriers, research will also be required to identify how best to combine interventions that are found to be effective. Higher linkage rates than ours have been observed in some studies that used counselling to facilitate linkage to care 43. However, most of these studies were prone to confounding, used counselling alongside other interventions 6, 7, 9, or were conducted in settings where linkage was high even in the absence of any intervention 37.

Consistent with previous studies in similar settings 42, 44, uptake of repeat HCT among HIV‐negative individuals was high. Previous HCT may reduce the psychological stress associated with testing hence making it easier to accept repeat HCT. Counselling had no measurable effect on uptake of repeat HCT. This may partly be due to the high (79%) uptake of HCT in the control arm, and partly due to the small sample size.

Despite the strong overall intervention effect, outcomes varied substantially between clusters with some intervention clusters showing poorer outcomes than control clusters (Supplementary File). This may have been due to chance or interaction between the intervention and key cluster‐level characteristics such as access to health facilities. Variations in implementation of the intervention are unlikely to have affected response to intervention since the same counsellors worked across all clusters.

The study had a number of strengths. It is the first randomized trial to demonstrate that counselling provided after referral substantially increases linkage to care among HIV‐positive persons identified through HBHCT in SSA. The trial was conducted under real‐world conditions in a relatively large geographical area with facilities that provided standard services. Participation and retention rates were high and did not differ by treatment arm thereby reducing risk of selection bias. The cluster‐randomized design further minimized selection bias, and ensured that the risk of contamination between treatment arms, and consequently that of diluting intervention effects, was low. Referrals were tracked to verify outcomes for all participants who reported having linked to care. This minimized the risk of bias that would have resulted from reliance on self‐reported data or incomplete tracking of referrals.

The study also had some limitations. First, it was an open‐label study: knowledge of the interventions may have influenced participants’ decisions to remain in the study. However, since there was no differential loss to follow‐up between arms, this is unlikely to have been a major issue. Second, whereas registering for care is a critical step in the HIV care cascade 27, 45, it may not result in sustained engagement in care 46. Therefore, more research may be necessary to demonstrate the effectiveness of counselling on retention in care and other long‐term care outcomes. Third, the counselling sessions and collection of outcome data were conducted by the same person for some participants. This may have increased the risk of reporting bias. In general, reporting bias is unlikely to have been a major issue since only data on adherence to CTXp were based on self‐reports. Fourth, the observed *k* was higher than that assumed for the sample size estimation implying that the trial could potentially have been underpowered.

## 5 Conclusions

The results of this trial suggest that counselling strongly increases linkage to care and uptake of other services including ART among HIV‐positive adults identified through HBHCT. In settings such as ours, counselling may enhance efforts aimed at achieving the second UNAIDS target (i.e. receipt of ART for 90% of the people who know their HIV status) 47 and should be considered for scaling up.

## Competing interests

The authors have no competing interests to declare.

## Authors’ contributions

All authors contributed to the conception and design of the study; E.R. conducted the study; A.K. provided technical oversight; E.R. and K.B. did the data analysis; E.R., H.G. and K.B. developed the first draft. All authors participated in writing the manuscript and approved the final version.

## Funding

This study was jointly funded by the UK Medical Research Council (MRC) and the UK Department for International Development (DFID) under the MRC/DFID Concordat agreement and is also part of the EDCTP2 program supported by the European Union. The International AIDS Vaccine Initiative provided funds for HIV test kits. The Department of Infectious Disease Epidemiology, London School of Hygiene & Tropical Medicine provided funds to cover CD4 count tests.

## Supporting information


**Table S1**. Proportions of HIV‐positive participants linking to care in each cluster
**Table S2**. Cluster‐level rates of linkage to care among HIV‐positive participants
**Table S3**. Cluster‐level rates of obtaining CD4 count results among HIV‐positive participants
**Table S4**. Cluster‐level rates of ART initiation among HIV‐positive participants
**Table S5**. Proportions of HIV‐positive participants adhering to cotrimoxazole prophylaxis (CTXp) in each cluster
**Table S6**. Proportions of HIV‐negative participants undergoing repeat HIV testing at 6 months in each cluster .
