# Supplementary material for: Brief counselling after home‐based HIV counselling and testing strongly increases linkage to care: a cluster‐randomized trial in Uganda
Source: J Int AIDS Soc. 2017 Oct 20;20(2):e25014. doi: 10.1002/jia2.25014 (PMC5810339; doi:10.1002/jia2.25014)
Supplement: Supplementary file 1 — Table S1. Proportions of HIV‐positive participants linking to care in each cluster Table S2. Cluster‐level rates of linkage to care among HIV‐positive participants Table S3. Cluster‐level rates of obtaining CD4 count results among HIV‐positive participants Table S4. Cluster‐level rates of ART initiation among HIV‐positive participants Table S5. Proportions of HIV‐positive participants adhering to cotrimoxazole prophylaxis (CTXp) in each cluster Table S6. Proportions of HIV‐negative participants undergoing repeat HIV testing at 6 months in each cluster . [file JIA2-20-e25014-s001.pdf]

Additional file 1: Cluster-level summaries

Table 1: Proportions of HIV-positive participants linking to care in each cluster

Intervention arm			Control arm		
**Cluster**	**Number enrolled**	**Number linked (%)**	**Cluster**	**Number enrolled**	**Number linked (%)**
**3**	6	2 (33.3)	**8**	15	4 (26.7)
**13**	7	2 (28.6)	**9**	12	6 (50.0)
**17**	17	10 (58.8)	**19**	4	2 (50.0)
**20**	12	5 (41.7)	**22**	6	1 (16.7)
**29**	8	3 (37.5)	**25**	10	5 (50.0)
**33**	8	5 (62.5)	**37**	11	5 (45.5)
**35**	9	2 (22.2)	**43**	6	0 (0.0)
**38**	6	4 (66.7)	**46**	6	2 (33.3)
**52**	7	6 (85.7)	**50**	15	2 (13.2)
**53**	13	9 (69.2)	**62**	17	9 (52.9)
**54**	26	11 (42.3)	**65**	17	8 (47.1)
**73**	7	3 (42.9)	**70**	9	4 (44.4)
**74**	13	8 (61.5)	**71**	17	3 (17.6)
**75**	10	6 (60.0)	**72**	8	0 (0.0)

Table 2: Cluster-level rates of linkage to care among HIV-positive participants

Intervention arm					Control arm				
**Cluster**	**Number enrolled**	**Number linked**	**PM**	**Rate/100 PM**	**Cluster**	**Number enrolled**	**Number linked**	**PM**	**Rate/100 PM**
**3**	6	2	21.5	9.3	**8**	15	4	64.1	6.2
**13**	7	2	30.7	6.5	**9**	12	6	36.1	16.6
**17**	17	10	56.2	17.8	**19**	4	2	9.0	22.3
**20**	12	5	44.4	11.3	**22**	6	1	26.8	3.7
**29**	8	3	28.8	10.4	**25**	10	5	29.6	16.9
**33**	8	5	19.2	26.0	**37**	11	5	37.5	13.3
**35**	9	2	36.1	5.5	**43**	6	0	32.1	0.0
**38**	6	4	12.4	32.4	**46**	6	2	23.2	8.6
**52**	7	6	12.8	46.8	**50**	15	2	74.8	2.7
**53**	13	9	36.4	24.7	**62**	17	9	47.2	19.0
**54**	26	11	101.9	10.8	**65**	17	8	58.6	13.6
**73**	7	3	24.9	12.0	**70**	9	4	36.0	11.1
**74**	13	8	36.2	22.1	**71**	17	3	70.6	4.2
**75**	10	6	31.0	19.3	**72**	8	0	44.1	0.0

PM: person-months

Table 3: Cluster-level rates of obtaining CD4 count results among HIV-positive participants

Intervention arm					Control arm				
**Cluster**	**Number Enrolled**	**Number receiving CD4 counts**	**PM**	**Rate/ 100 PM**	**Cluster**	**Number Enrolled**	**Number receiving CD4 counts**	**PM**	**Rate/ 100 PM**
**3**	6	2	21.5	9.3	**8**	15	3	73.2	4.1
**13**	7	1	34.5	2.9	**9**	12	6	38.0	15.8
**17**	17	8	67.4	11.9	**19**	4	1	14.6	6.9
**20**	12	5	47.8	10.5	**22**	6	1	29.0	3.4
**29**	8	3	30.1	10.0	**25**	10	4	37.6	10.7
**33**	8	5	21.6	23.2	**37**	11	4	42.8	9.4
**35**	9	2	36.1	5.5	**43**	6	0	32.1	0.0
**38**	6	4	14.1	28.3	**46**	6	0	33.4	0.0
**52**	7	4	26.1	15.3	**50**	15	2	77.1	2.6
**53**	13	7	47.5	14.7	**62**	17	9	48.4	18.6
**54**	26	11	107.8	10.2	**65**	17	6	70.4	8.5
**73**	7	3	26.2	11.5	**70**	9	3	43.9	6.8
**74**	13	6	47.2	12.7	**71**	17	1	79.1	1.3
**75**	10	6	32.6	18.4	**72**	8	0	44.1	0.0

PM: person-months

Table 4: Cluster-level rates of ART initiation among HIV-positive participants

**Intervention arm**					**Control arm**				
**Cluster**	**Number enrolled**	**Number initiating ART**	**PM**	**Rate/100 PM**	**Cluster**	**Number enrolled**	**Number initiating ART**	**PM**	**Rate/100 PM**
**3**	6	1	27.2	3.7	**8**	15	4	68.1	5.9
**13**	7	1	35.1	2.9	**9**	12	5	42.5	11.8
**17**	17	6	71.7	8.4	**19**	4	2	9.0	22.3
**20**	12	3	56.5	5.3	**22**	6	1	31.9	3.1
**29**	8	3	32.0	9.4	**25**	10	4	36.3	11.0
**33**	8	5	22.9	21.8	**37**	11	3	50.5	5.9
**35**	9	2	36.1	5.5	**43**	6	0	32.1	0.0
**38**	6	3	17.9	16.7	**46**	6	2	23.2	8.6
**52**	7	3	30.5	9.9	**50**	15	1	79.9	1.3
**53**	13	6	53.2	11.3	**62**	17	8	50.0	16.0
**54**	26	6	127.0	4.7	**65**	17	5	76.0	6.6
**73**	7	2	32.6	6.1	**70**	9	4	38.9	10.3
**74**	13	6	45.0	13.3	**71**	17	1	79.1	1.3
**75**	10	3	42.0	7.1	**72**	8	0	44.1	0.0

PM: person-months

Table 5: Proportions of HIV-positive participants adhering to cotrimoxazole prophylaxis (CTXp) in each cluster

Intervention arm			Control arm		
Cluster	Number enrolled	Number adhering to CTXp (%)	Cluster	Number enrolled	Number adhering to CTXp (%)
**3**	6	1 (16.7)	**8**	15	3 (20.0)
**13**	7	1 (14.3)	**9**	12	6 (50.0)
**17**	17	10 (58.8)	**19**	4	1 (25.0)
**20**	12	4 (33.3)	**22**	6	0 (0.0)
**29**	8	3 (37.5)	**25**	10	5 (50.0)
**33**	8	5 (62.5)	**37**	11	4 (36.4)
**35**	9	1 (11.1)	**43**	6	0 (0.0)
**38**	6	4 (66.7))	**46**	6	1 (16.7)
**52**	7	3 (42.9)	**50**	15	2 (13.3)
**53**	13	7 (53.8)	**62**	17	8 (47.1)
**54**	26	10 (38.5)	**65**	17	7 (41.2)
**73**	7	3 (42.9)	**70**	9	4 (44.4)
**74**	13	8 (61.5)	**71**	17	2 (11.8)
**75**	10	6 (60.0)	**72**	8	0 (0.0)

Table 6: Proportions of HIV-negative participants undergoing repeat HIV testing at 6 months in each cluster

Intervention arm			Control arm		
Cluster	Number enrolled	Number repeat testing (%)	Cluster	Number enrolled	Number repeat testing (%)
**3**	2	2 (100.0)	**8**	7	6 (85.7)
**13**	3	1 (33.3)	**9**	8	6 (75.0)
**17**	8	7 (87.5)	**19**	1	1 (100.0)
**20**	4	4 (100.0)	**22**	2	1 (50.0)
**29**	2	1 (50.0)	**25**	4	4 (100.0)
**33**	2	1 (50.0)	**37**	2	1 (50.0)
**35**	3	3 (100.0)	**43**	2	2 (100.0)
**38**	2	2 (100.0)	**46**	3	3 (100.0)
**52**	2	2 (100.0)	**50**	5	4 (80.0)
**53**	4	3 (75.0)	**62**	6	4 (66.7)
**54**	10	8 (80.0)	**65**	8	6 (75.0)
**73**	3	2 (66.7)	**70**	3	2 (66.7)
**74**	4	3 (75.0)	**71**	5	4 (80.0)
**75**	3	3 (100.0)	**72**	2	2 (100.0)

Review Only
